# Chenodeoxycholic Acid (CDCA) Promoted Intestinal Epithelial Cell Proliferation by Regulating Cell Cycle Progression and Mitochondrial Biogenesis in IPEC-J2 Cells

**DOI:** 10.3390/antiox11112285

**Published:** 2022-11-18

**Authors:** Lei Xu, Yanpin Li, Zixi Wei, Rong Bai, Ge Gao, Wenjuan Sun, Xianren Jiang, Junjun Wang, Xilong Li, Yu Pi

**Affiliations:** 1 Key Laboratory of Feed Biotechnology of Ministry of Agriculture and Rural Affairs, Institute of Feed Research, Chinese Academy of Agricultural Sciences, Beijing 100081, China; 2 Department of Business Economics, Wageningen University, 6700 EW Wageningen, The Netherlands; 3 State Key Laboratory of Animal Nutrition, College of Animal Science and Technology, China Agricultural University, Beijing 100193, China

**Keywords:** chenodeoxycholic acid (CDCA), cell proliferation, cell cycle, mitochondria, IPEC-J2 cells

## Abstract

Chenodeoxycholic acid (CDCA), a primary bile acid (BA), has been demonstrated to play an important role as a signaling molecule in various physiological functions. However, the role of CDCA in regulating intestinal epithelial cell (IEC) function remains largely unknown. Herein, porcine intestinal epithelial cells (IPEC-J2) were used as an in vitro model to investigate the effects of CDCA on IEC proliferation and explore the underlying mechanisms. IPEC-J2 cells were treated with CDCA, and flow cytometry and transcriptome analysis were adopted to investigate the effects and potential molecular mechanisms of CDCA on the proliferation of IECs. Our results indicated that adding 50 μmol/L of CDCA in the media significantly increased the proliferation of IPEC-J2 cells. In addition, CDCA treatment also hindered cell apoptosis, increased the proportion of G0/G1 phase cells in the cell cycle progression, reduced intracellular ROS, and MDA levels, and increased mitochondrial membrane potential, antioxidation enzyme activity (T-AOC and CAT), and intracellular ATP level (*p* < 0.05). RNA-seq results showed that CDCA significantly upregulated the expression of genes related to cell cycle progression (Cyclin-dependent kinase 1 (CDK1), cyclin G2 (CCNG2), cell-cycle progression gene 1 (CCPG1), Bcl-2 interacting protein 5 (BNIP5), etc.) and downregulated the expression of genes related to mitochondrial biogenesis (ND1, ND2, COX3, ATP6, etc.). Further KEGG pathway enrichment analysis showed that CDCA significantly enriched the signaling pathways of DNA replication, cell cycle, and p53. Collectively, this study demonstrated that CDCA could promote IPEC-J2 proliferation by regulating cell cycle progression and mitochondrial function. These findings provide a new strategy for promoting the intestinal health of pigs by regulating intestinal BA metabolism.

## 1. Introduction

Bile acids (BAs) are synthesized in the liver from cholesterol and stored in the gallbladder. After the dietary stimulus, they are released into the duodenum. Apart from the universally recognized roles in cholesterol homeostasis and fat digestion in the gastrointestinal tract, pieces of evidence have shown that BAs can also act as a key signaling molecule to regulate various biological effects, including glucose homeostasis [1,2], immunity [3,4], and cell proliferation and apoptosis [5,6], through acting on specific BA receptors, mainly farnesoid X receptor (FXR) and Takeda G-protein receptor 5 (TGR5), or other cellular signaling pathways.

Chenodeoxycholic acid (CDCA), also known as 3α,7α-dihydroxy-5β-cholan-24-oic acid, is one of the major primary BAs in the intestine of humans and animals. In pigs, hyocholic acid (HCA), CDCA, and cholic acid (CA) are the main primary BAs in the intestine, accounting for about 84%, 11%, and 4%, respectively [7]. CDCA has been applied in the clinical treatment of cholesterol gallstones in humans [8]. In addition, it has been shown that CDCA exerts regulatory effects on body metabolic functions [9], including obesity attenuation [10], brown adipose tissue activation [11], and insulin resistance improvement [12]. Moreover, it has been implicated that intraduodenal infusions of 15 mM CDCA were involved in stimulating the secretion of intestinal hormones such as glucagon-like peptide 1 (GLP-1) and cholecystokinin (CCK) in humans [13], that diet supplementation with 0.02% CDCA could improve intestinal morphology and barrier function in weaned piglets [14,15] and that 50 μM CDCA treatment alleviated intestinal inflammation in both porcine intestinal epithelial cells (IPEC-J2) and mouse models [16], suggesting that CDCA may elicit critical roles in regulating intestinal physiological function. However, the roles of CDCA in intestinal epithelial cell (IEC) proliferation remain unclear.

In the mammalian intestine, epithelial cells are renewed every 4–5 days [17]. Proliferation of intestinal epithelial cells plays an important role in normal intestinal growth and development [18]. In addition, cell proliferation is also involved in the restoration of intestinal epithelial tissue that has experienced inflammatory damage [19]. Therefore, cell proliferation is necessary for intestinal mucosal renewal in normal and diseased states. In pigs, especially in neonate piglets, promoting intestinal development and enhancing intestinal epithelial function are necessary for improving health [20]. However, several investigators have reported that BAs have different effects on the proliferation of IECs, including stimulatory effects [5,21], and inhibitory effects [22,23]. To date, the effects of CDCA on the proliferation of IECs and the possible underlying mechanism remain largely unknown.

IPEC-J2 is a non-transformed porcine intestinal epithelial cell line that originated from the jejunal epithelium of neonatal un-suckled piglets. These cells are morphologically and functionally similar to primary IECs [24]. Therefore, this study was designed to investigate the effects of CDCA on the proliferation of IECs using the IPEC-J2 model. In addition, cell transcriptomics was further used to explore the possible underlying mechanism. Our data showed that CDCA could promote IEC proliferation by regulating cell cycle progression and mitochondrial biogenesis.

## 2. Materials and Methods

### 2.1. Cell Culture

The IPEC-J2 cells were kindly supplied by Dr. Guoyao Wu’s laboratory (Texas A&M University, College Station, TX, USA). Cells were cultured in Dulbecco’s Modified Eagle Medium/F12 (DMEM/F12, Thermo Fisher Scientific, Waltham, MA, USA) supplemented with 5% fetal bovine serum (FBS, Thermo Fisher Scientific, Waltham, MA, USA) and 1% penicillin-streptomycin (Thermo Fisher Scientific, Waltham, MA, USA). They were grown at 37 °C in a humidified incubator with 5% CO_2_. The medium was replaced every other day. We used passage 12–15 cells in these studies.

### 2.2. Selection of CDCA Concentration

To select the optimal concentration of CDCA (≥98%, Shanghai Yuanye Bio-Technology Co., Ltd., Shanghai, China), IPEC-J2 cells were seeded in 96-well plates (Corning Incorporated, Corning, NY, USA) with a density of 1.5 × 10^5^ cells/mL (100 μL per well). After 24 h of incubation, cells were treated with different concentrations of CDCA (0, 10, 25, 50, 100, 200, and 400 μM) for another 24 h. Cell viability was measured using a cell counting kit (CCK-8, MedChemExpress, Monmouth Junction, NJ, USA) according to the manufacturer’s protocol. In brief, cells were incubated with 10% CCK-8 reagent for 3 h at 37 °C, and then an Epoch microplate spectrophotometer (BioTek Instruments, Incorporated, Winooski, VT, USA) was used to test the absorbance (A) at 450 nm. The calculation formula of cell viability was as follows: Cell viability = [A (treatment group) − A (blank group)]/[A (control group) − A (blank group)] × 100%. The cell viability of the control group was considered to be 100%. In this study, 50 μM CDCA was selected to carry out the following experiments, since it had higher cell viability.

### 2.3. Proliferation Assay

IPEC-J2 cells were seeded in 96-well plates (Corning Incorporated, Corning, NY, USA) with a density of 1.5 × 10^5^ cells/mL (100 μL per well). After 24 h of incubation, cells were treated with 50 μM CDCA for another 24 h. Cells were incubated with 5-ethynyl-2′-deoxyuridine (EdU) for 2 h before staining. Cell proliferation was detected using Cell-Light EdU Cell Proliferation Detection Kit (Ribobio, Guangzhou, China) according to the manufacturer’s protocol. The percentage of proliferative cells was determined by quantitation of EdU-positive cells using an Olympus fluorescent microscope (Olympus, Tokyo, Japan).

### 2.4. Measurement of Apoptosis, Cell Cycle, Mitochondrial Membrane Potential, and Intracellular Reactive Oxygen Species

Apoptosis ratio, cell cycle, mitochondrial membrane potential, and intracellular reactive oxygen species (ROS) levels were detected by flow cytometry (FCM). In brief, IPEC-J2 cells were seeded in 6-well plates and treated with or without 50 μM CDCA for 24 h. The cells were then harvested and measured for apoptosis using an annexin V-FITC/PI apoptosis detection kit (Beijing 4A Biotech Co., Ltd., Beijing, China), cell cycle using a PI cell cycle analysis kit (Beijing 4A Biotech Co., Ltd., Beijing, China), mitochondrial membrane potential using a JC-1 staining kit (Beyotime Biotechnology, Haimen, China), and intracellular ROS level using a ROS active oxygen detection kit based on DCFH-DA fluorescent probe (Beyotime Biotechnology, Haimen, China) according to the corresponding manufacturer’s instructions. The stained cells were characterized by a high-sensitivity flow cytometer (Beckman Coulter CytoFlex S, Krefeld, Germany).

### 2.5. Determination of Cellular Antioxidant Indices and ATP Level

IPEC-J2 cells were seeded in 6-well plates (Corning Incorporated, Corning, NY, USA) with a density of 1.5 × 10^5^ cells/mL (2 mL per well). After 24 h of incubation, cells were treated with or without 50 μM CDCA for another 24 h. Subsequently, the cells were lysed by RIPA buffer (Thermo Fisher Scientific, Waltham, MA, USA) at 4 °C for 30 min, and the supernatant was collected after centrifugation at 13,000× *g* at 4 °C for 30 min. The total antioxidant capacity (T-AOC), catalase (CAT) activity, and malondialdehyde (MDA) and adenosine triphosphate (ATP) levels were detected using corresponding assay kits (Nanjing Jiancheng Bioengineering Institute, Nanjing, China) according to the manufacturer’s protocols. The total protein concentration was determined using a BCA protein assay kit (Nanjing Jiancheng Bioengineering Institute, Nanjing, China).

### 2.6. RNA Extraction, Library Preparation, Sequencing, and Data Analysis

For RNA sequencing, IPEC-J2 cells in 6-well plates were treated with or without CDCA (50 μM) for 24 h. Total RNA was extracted from the cell sample using TRIzol^®^ Reagent according to the manufacturer’s instructions (Invitrogen, Carlsbad, CA, USA). Then, RNA quality was assessed using a 2100 Expert Bioanalyzer (Agilent Technologies, Santa Clara, CA, USA) and quantified using the ND-2000 (NanoDrop Technologies, Wilmington, DE, USA). The high-quality RNA samples (OD 260/280 = 1.8–2.2, OD 260/230 ≥ 1.0, RIN ≥ 6.5, total RNA ≥ 1.0 μg, concentration ≥ 35 ng/μL) were used to construct the sequencing library by Majorbio Biotech (Shanghai, China) on an Illumina Novaseq 6000. Short sequence reads were analyzed on the Majorbio I-Sanger Cloud Platform (https://cloud.majorbio.com/) (accessed on 21 September 2022). To identify differentially expressed genes (DEGs) between two different samples, gene expression was expressed in transcripts per million (TPM). RNA-seq by expectation-maximization (RSEM) was used to quantify the gene abundances [25]. R statistical package software EdgeR (Empirical Analysis of Digital Gene Expression in R) was utilized for DEG analysis [26], and the resulting *p*-values were further processed by FDR correction with the Benjamini–Hochberg (BH) method. GO functional enrichment and KEGG pathway analysis were carried out by Goatools [27] and KOBAS [28]. The protein–protein interaction (PPI) network was forecasted based on the STRING online database (http://www.string-db.org/) (accessed on 9 October 2022) [29]. The PPI network of DEGs in IPEC-J2 was evaluated, and interactions with an overall score > 0.4 were considered statistically significant. The key gene in the PPI network was investigated topologically by using Network Analyzer plugin, which is well-integrated into Cytoscape Software. Three centrality methods, including degree, closeness, and betweenness centrality, were used to explore the key genes in the PPI network. All of the RNA sequencing data were deposited in NCBI’s Gene Expression Omnibus (GEO) under the accession number GSE214153.

### 2.7. RNA Extraction and RT-qPCR

Total RNA was extracted from IPEC-J2 cells using TRIzol (Takara Bio Inc., Dalian, China), and 1 μg RNA was reverse-transcribed using PrimeScript^®^ RT Reagent Kit with cDNA Eraser (Takara Bio Inc., Dalian, China). RT-qPCR was performed with gene-specific primers (Table S1) and an SYBR Green master mix on a CFX96 real-time PCR system (Bio-Rad, Hercules, CA, USA). Relative fold changes of gene expression were calculated using the cycle threshold (Ct) method and β-actin or GAPDH as a reference gene, as previously described.

### 2.8. mtDNA Copy Number Determination

Total genomic DNA was extracted from the IPEC-J2 cell samples with a DNA tissue kit (QIAGEN, Hilden, German). RT-PCR was performed with mtDNA-specific primer (D-loop region) (Table S1) and an SYBR Green master mix on a CFX96 real-time PCR system (Bio-Rad, Hercules, CA, USA). Relative fold changes of gene expression were calculated using the 2^−(ΔΔCt)^ method and nuclear DNA (β-actin) as a reference gene, as previously described [30].

### 2.9. Statistical Analysis

All statistical significance was assessed by the independent sample *t*-test using SPSS (SPSS 20 software, IBM, Armonk, NY, USA). All results were expressed as means ± SEM. Differences were considered statistically significant if *p* < 0.05.

## 3. Results

### 3.1. Effects of Different Doses of CDCA on Cell Viability of IPEC-J2 Cells

**Figure 1:**
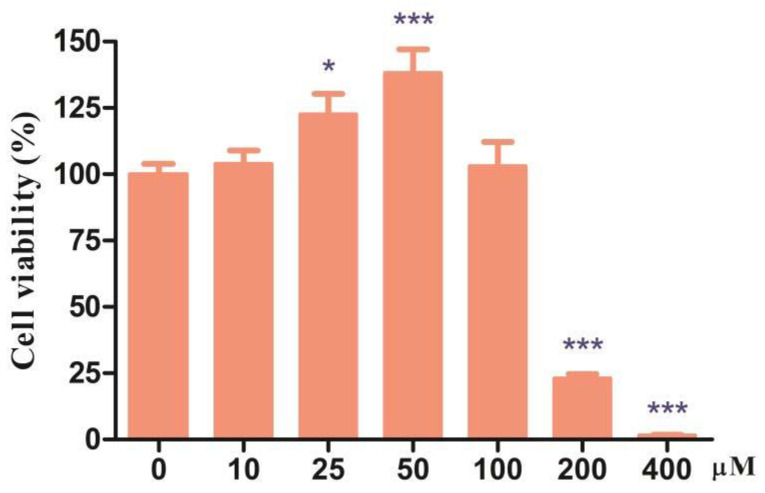
Effects of CDCA on the viability of IPEC-J2 cells (*n* = 6). Cells were seeded at a density of 1.5 × 10^5^/well in 96-well plates. Values are presented as mean ± SEM and expressed as a percentage decrease or increase in cell viability. * *p* < 0.05, *** *p* < 0.001 compared with the CON group.

To investigate the effect of CDCA on the porcine intestinal epithelial cells, IPEC-J2 cells were treated with CDCA at various concentrations for 24 h. As shown in Figure 1, compared to the CON group, the CDCA-treated group significantly increased cell viability at 25 and 50 μM (*p* < 0.05). However, when the concentration of CDCA was higher than 200 μM, the cell viability was significantly decreased (*p* < 0.05). These results indicated that an appropriate concentration of CDCA is conducive to cell proliferation. Therefore, 50 μM CDCA was selected to carry out the following experiments because of higher cell viability at this concentration.

### 3.2. CDCA Reduces Apoptosis, Improves Mitochondrial Function, and Regulates the Cell Cycle

**Figure 2:**
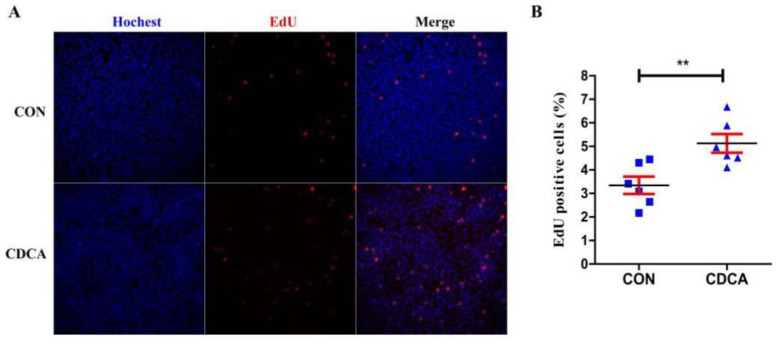
Effects of CDCA on the proliferation of IPEC-J2 cells (*n* = 6). The representative EdU staining plots of IPEC-J2 cells (**A**) and the proportion of EdU-positive cells (**B**). Cells were seeded at a density of 1.5 × 10^5^/well in 96-well plates. Values are presented as mean ± SEM. ** *p* < 0.01 compared with the CON group.

**Figure 3:**
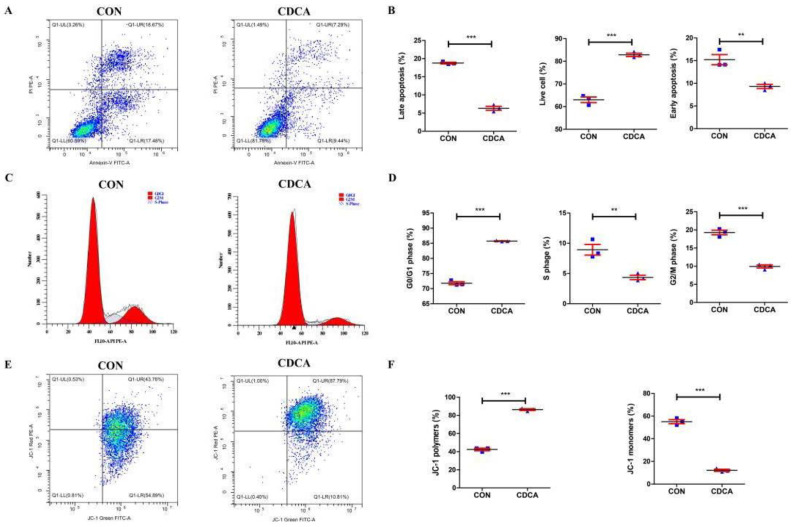
Effects of CDCA on cell apoptosis, cell cycle, and mitochondrial membrane potential of IPEC-J2 cells (*n* = 3). The representative flow cytometry (FCM) plots of cell apoptosis (**A**) as well as the proportion of late apoptosis cells, live cells, and early apoptosis cells (**B**). The representative FCM plots of the cell cycle (**C**) as well as the proportion of cells in G0/G1, S, and G2/M phases (**D**); The representative FCM plots of cell mitochondrial membrane potential (**E**) as well as the proportion of cells with JC-1 polymers (high membrane potential) and JC-1 monomers (low membrane potential) (**F**). Values are presented as mean ± SEM. ** *p* < 0.01, *** *p* < 0.001 compared with the CON group.

To verify the effect of CDCA on the physiological function of porcine intestinal epithelial cells, the cell proliferation was further analyzed by EdU assays, and the apoptotic ratio, mitochondrial membrane potential, and cell cycle were further analyzed by FCM. Consistent with the results for cell viability, 50 μM CDCA treatment for 24 h caused significantly promoted cell proliferation (Figure 2A,B), a prominent increase in the proportion of live cells, and a decrease in both the early and late apoptotic cells compared with those in the CON group (Figure 3A,B). Growth increase can be attributed to cell cycle enhancement. Thus, we wondered whether CDCA could modulate cell cycle progression. As a result, CDCA treatment in IPEC-J2 cells caused a significant accumulation of cells in the G0/G1 phase and markedly decreased the proportion of cells in the S- and G2/M-phases compared with the CON group (Figure 3C,D). These results indicate that CDCA could promote cell cycle progression, which may be conducive to cell proliferation. Mitochondria are important organelles that regulate cell survival; their function can be reflected by the mitochondrial membrane potential. Thus, the mitochondrial membrane potential of IPEC-J2 cells was further analyzed using JC-1 staining. JC-1 is capable of selectively entering the mitochondria, where it forms monomers (lower membrane potential) and polymers (higher membrane potential). After IPEC-J2 cells were treated with 50 μM CDCA for 24 h, the percentage of cells with polymers increased, and cells with monomers decreased (Figure 3E,F), suggesting the mitochondrial function in CDCA-induced IPEC-J2 cells was better than that in CON cells.

### 3.3. CDCA Could Enhance Redox Balance in IPEC-J2 Cells

**Figure 4:**
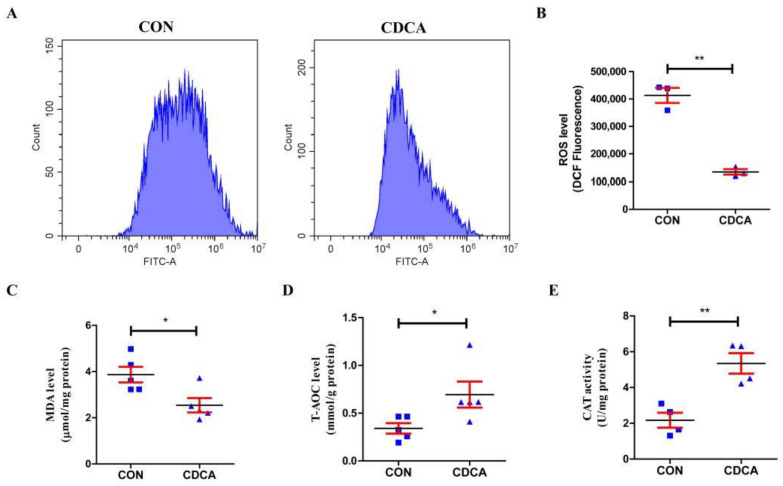
Effects of CDCA on oxidative stress and oxidation resistance of IPEC-J2 cells. The representative flow cytometry plots (**A**) of cell intracellular ROS level (*n* = 3) (**B**); MDA level (**C**), T-AOC activity (**D**), and CAT activity (**E**) of IPEC-J2 cells treated with CDCA (*n* = 5). Values are presented as mean ± SEM. * *p* < 0.05, ** *p* < 0.01 compared with the CON group. ROS, reactive oxygen species; MDA, malondialdehyde; T-AOC, total antioxidant capacity; CAT, catalase.

ROS production is one of the important indicators of cell health status in mammalian cells. Therefore, we further examined the level of intracellular ROS in IPEC-J2 cells with 50 μM CDCA or without CDCA treatment for 24 h. As shown in Figure 4, CDCA treatment significantly decreased intracellular ROS levels (Figure 4A,B). In addition, CDCA also significantly reduced the MDA level (Figure 4C) and increased T-AOC (Figure 4D) and CAT activity (Figure 4E) in IPEC-J2 cells. These results indicate that CDCA could enhance the redox balance by increasing antioxidant enzyme activity and decreasing intracellular ROS and MDA production.

### 3.4. The Transcriptome Response to CDCA Treatment in IPEC-J2 Cells

Further RNA-seq analysis of IPEC-J2 cells after CDCA treatment was conducted to explore the mechanisms of the effects of CDCA on cell function. In the present study, a total of six cDNA libraries from the CON and CDCA groups were established and sequenced. The RNA-sequencing of six samples obtained total raw paired-end reads of around 383.9 million (Table S2). After quality filtering, each sample remained with high-quality clean reads of approximately 63.3 million, ranging from 60.1 to 68.5 million. The high-quality clean reads were further mapped to the reference sequences, and the alignment results were assessed to achieve secondary quality control and estimate whether the reference was reasonable. After alignment, 95.47–95.96% and 95.62–95.74% of the clean reads were successfully mapped to the genome; 6.84–8.92% and 4.83–4.91% of the clean reads were found for multiple mapped reads; 86.55–88.94% and 90.71–90.91% of the clean reads were found for uniquely mapped reads in CON and CDCA groups, respectively (Table S2). Outlier box plots showed that all of the samples had a similar data distribution and none was considered an outlier (Figure S1A). The correlation analysis between samples showed that biological replicates from the two groups could be well distinguished and the experimental design was reasonable (Figure S1B). These results confirmed the high reproducibility of the RNA-sequencing data.

**Figure 5:**
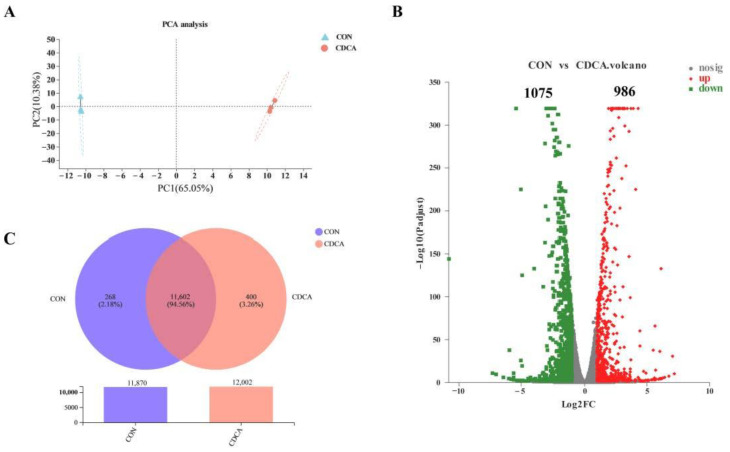
Effects of CDCA on the DEGs of IPEC-J2 cells (*n* = 3). PCA plot of the transcriptional profiles between the two groups (**A**). Volcano plot of DEGs between CON and CDCA groups (**B**). The red, green, and gray dots indicate the significantly upregulated, significantly downregulated, and unchanged genes, respectively. The Venn diagram showed the number of specifically expressed genes in each group, as well as the overlapping co-expressed genes between CON and CDCA groups (**C**). DEGs, differentially expressed genes.

PCA plot showed an obvious shift in gene expression with CDCA treatment (Figure 5A). The volcano map could indicate the whole DEG distribution. Thus, the volcano map was used to show the overall situation of the DEGs. Compared with the CON group, the CDCA group significantly upregulated 986 genes and downregulated 1075 genes (fold change > 2; FDR < 0.05) (Figure 5B). Based on these results, we selected 11 downregulated DEGs, 3 upregulated DEGs and 6 unchanged genes for further validation by RT-qPCR. The results showed that the gene expression observed by RNA-seq was similar to that by RT-qPCR analysis, suggesting that the results of RNA-seq were reliable (Figure S2). The Venn plot indicated that 11,602 genes were common between the two groups, and 268 and 400 genes were uniquely expressed in the CON and CDCA groups, respectively (Figure 5C).

**Figure 6:**
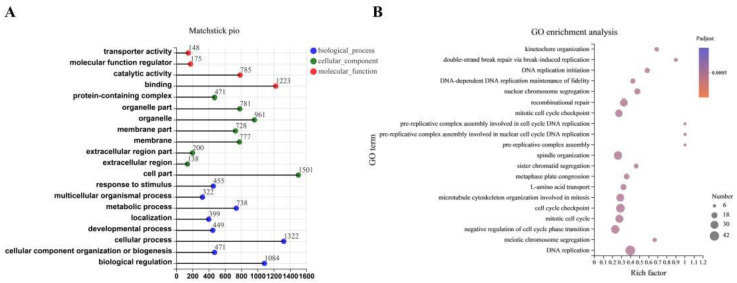
Effects of CDCA on GO functional annotation and enrichment analysis of IPEC-J2 cells. Matchstick Pio of GO functional annotation results for CON and CDCA groups (**A**). Scatter plot of GO functional enrichment results for CON and CDCA groups (**B**). GO, gene ontology.

**Figure 7:**
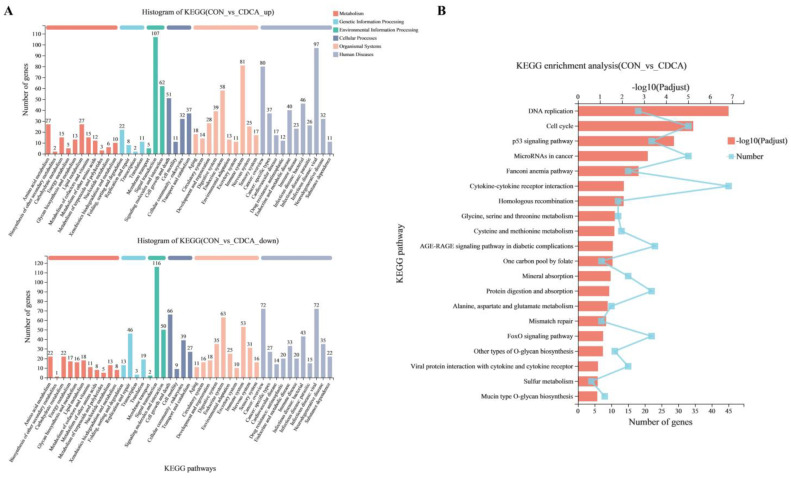
Effects of CDCA on KEGG functional annotation and enrichment analysis of IPEC-J2 cells. Histogram of KEGG functional annotation results for CON and CDCA groups (**A**). Scatter plot of KEGG enrichment results for CON and CDCA groups (**B**). KEGG, Kyoto Encyclopedia of Genes and Genomes.

The DEGs were subjected to GO functional category analysis, followed by GO enrichment analysis. All of the DEGs were enriched in three main functional categories (top 20), including biological process (BP), cellular components (CC), and metabolic function (MF) (Figure 6A). At the BP level, the uppermost enrichment factor of DEG enrichment in GO terms was the cellular process. In the CC category, most of the DEGs were classified into cell parts. At the MF level, most of the DEGs were enriched in binding. The results of GO enrichment (top 20) are shown in Figure 6B. CDCA treatment markedly affected the GO terms (FDR < 0.05), including cell cycle checkpoint, mitotic cell cycle, DNA replication, and mitotic cell cycle checkpoint. To better understand the functional changes in the IPEC-J2 cells with CDCA treatment, the DEGs were subjected to the KEGG database for pathway enrichment analysis. KEGG pathways have six categories, namely cellular processes, genetic information processing, metabolism, environmental information processing, organismal systems, and human diseases. The significant changes in the KEGG pathways after CDCA treatment of IPEC-J2 cells are shown in Figure 7A,B. Furthermore, KEGG pathway enrichment analysis results showed that CDCA treatment significantly enriched KEGG pathways, including DNA replication, cell cycle, and p53 signaling pathway (Figure 7C).

**Figure 8:**
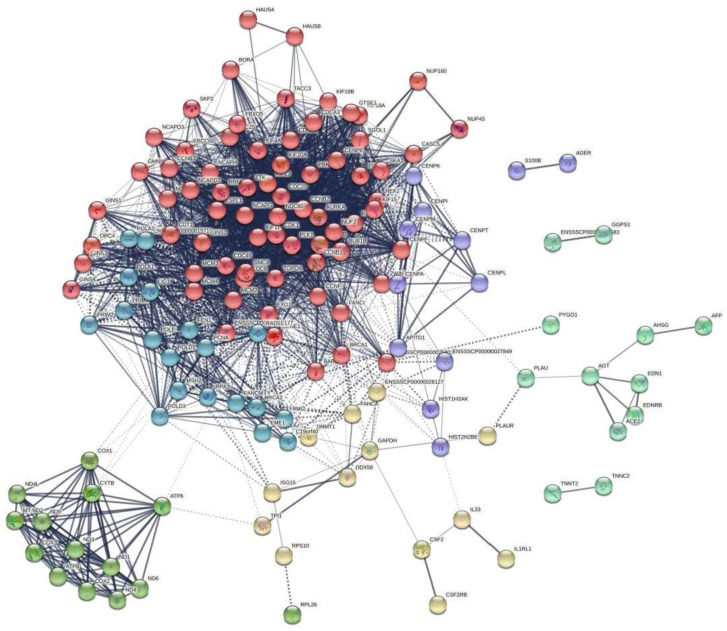
The protein–protein interaction (PPI) network analysis was based on the DEGs after CDCA treatment. The network was set up according to the string online tool. Nodes represent genes, and the weights of the edges represent how specifically two genes are associated together. Of the six clusters (represented by six colors), the red cluster includes genes related to the cell cycle, and the lime green cluster includes genes related to mitochondrial biogenesis. The detailed information for node attributes, node degree, and clusters of the PPI network is shown in Tables S3–S5, respectively.

To systemically analyze the functions of the DEGs in IPEC-J2 cells, we mapped the DEGs to PPI data and obtained some PPI networks. As shown in Figure 8, a total of 300 relationships (edges) between 141 genes (nodes) were identified. Node centrality analysis and the node genes with a ranked degree both showed that CDK1 formed a network with the highest degree (Figure S3, Tables S3, S4, S6). The CDK1 protein plays key roles in multiple cellular activities including the cell cycle, DNA replication, and apoptotic process. In addition, among the six clusters in the PPI network (Table S5), cell cycle-related and mitochondrial biogenesis-related genes were clustered, suggesting the cell cycle and mitochondrial function were modulated by CDCA treatment.

### 3.5. Cell Apoptosis, Cell Cycle, and Mitochondrial Biogenesis-Related Gene Expression Regulated by CDCA Treatment in IPEC-J2 Cells

**Figure 9:**
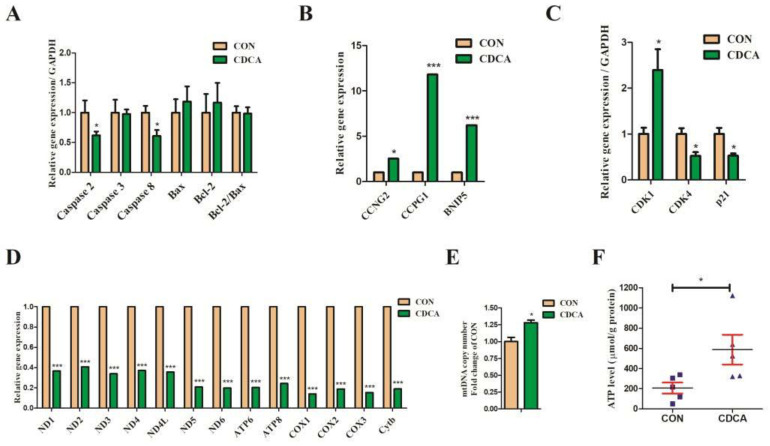
The genes related to cell apoptosis, cell cycle, and mitochondrial content expression are regulated by CDCA treatment. Relative expression of genes related to cell apoptosis (**A**). Relative expression of genes related to cell cycle (**B**,**C**). Relative expression of mtDNA-encoded gene, NADH dehydrogenase subunits (ND1, ND2, ND3, ND4, etc.), cytochrome b (Cytb), cytochrome c oxidase subunits (COX1, COX2, and COX3), and ATP synthase F0 subunits (ATP6 and ATP8) by RNA-seq (**D**). The copy number of mtDNA (**E**). The level of cellular ATP regulated by CDCA treatment in IPEC-J2 cells (**F**). * *p* < 0.05, *** *p* < 0.001 compared with the CON group. Bax, BCL2-associated X; CCNG2, cyclin G2; CCPG1, cell-cycle progression gene 1; Bcl-2, B-cell lymphoma-2; BNIP5, Bcl-2 interacting protein 5; CDK, Cyclin-dependent kinase; ND, NADH dehydrogenase subunit; COX, cytochrome c oxidase subunit; Cytb, cytochrome b; ATP, ATP synthase F0 subunit.

As for the cell apoptosis-related genes, Caspase 2 and Caspase 8 gene expression were significantly downregulated by CDCA, while Caspase 3, Bax, and Bcl-2 gene expression and the ratio of Bcl-2/Bax were unchanged by CDCA (Figure 9A). As for cell cycle-related gene expression, Cyclin-dependent kinase 1 (CDK1), cyclin G2 (CCNG2), cell-cycle progression gene 1 (CCPG1), and Bcl-2 interacting protein 5 (BNIP5) were significantly upregulated by CDCA, while CDK4 and p21 were significantly downregulated by CDCA (Figure 9B,C). As for mitochondrial biogenesis-related genes, the relative expressions of the mtDNA-encoded gene, NADH dehydrogenase subunits (ND1, ND2, ND3, ND4, etc.), cytochrome b (Cytb), cytochrome c oxidase subunits (COX1, COX2, and COX3), and ATP synthase F0 subunits (ATP6 and ATP8) were significantly downregulated by CDCA (Figure 9D). In addition, the copy number of mtDNA (Figure 9E) and level of intracellular ATP increased after CDCA treatment (*p* < 0.05) (Figure 9F), suggesting that CDCA promoted cell proliferation in IPEC-J2 cells possibly by regulating both the cell cycle progression and mitochondrial biogenesis.

## 4. Discussion

CDCA is one of the major primary BAs in the intestine of humans and animals. CDCA has been applied in the clinical treatment of cholesterol gallstones in humans [8] and also has the function of regulating body metabolic functions [9], intestinal hormone (GLP-1 and CCK) secretion [13], and anti-inflammatory action [16]. However, the role of CDCA in IEC proliferation remained unclear. In this study, for the first time, we provided evidence that an appropriate level (50 μM) of CDCA could promote cell proliferation accompanied by an acceleration of cell cycle progression in S and G2/M phases as well as improve mitochondrial function and the reduction of intracellular ROS production in IPEC-J2 cells.

Cell-cycle progression is the basis of cell proliferation, which is regulated by cell cycle-related proteins, including cyclins and cyclin-dependent kinases (CDKs) [31]. Cyclin D1, as a key regulator, can lead to promoting the G1 to S phase. Cyclin D1 binding with CDK 4/6 plays a key role in the G1 to S checkpoint [32]. CDK1 is one of the critical determinants for the transition from the G2 phase into mitosis [33]. Inhibition of CDKs can lead to cell-cycle suspension at different phases or checkpoints [34]. The p21 encodes a universal inhibitor of CDKs [35]. Indeed, in the present study, CDCA significantly upregulated gene expression of CDK1 and downregulated gene expression of p21, accompanied by a reduced proportion of cells in the G2/M phase. In addition, there is evidence demonstrating the role of CCNG2 in G2/M regulation [36]. The previous study proved that CCNG2 knock-down by siRNA could increase the G2/M phase proportion in LSCC cell lines [37]. This is consistent with our findings that the gene expression of CCNG2 in IPEC-J2 cells was upregulated by CDCA treatment, leading to a significant decrease in the G2/M phase proportion. Thus, there is a chance that CDCA accelerated the cell cycle progression by promoting cell transition from the G2 phase into mitosis.

The p53 signaling pathway can affect DNA replication fidelity and cell division when the cell responds to extrinsic and intrinsic stresses [38]. p21 is a key target gene of the p53 signaling pathway and has been proven to regulate cell cycle arrest in the event of DNA damage [39]. Our study showed that the p53 signaling pathway was significantly enriched and gene expression of p21 was downregulated by CDCA treatment, which consequently inhibited cell cycle arrest and promoted cellular proliferation.

Mitochondria are critical targets for BA toxicity at the cellular level [40,41,42]. It has been well-characterized that BAs degrade mitochondrial function [41,43]. The mitochondrial membrane potential collapse, mitochondrial permeabilization and swelling, mitochondrial ATP biosynthesis impairment, and release of cell death mediators are attributed to BA cytotoxicity [41,44,45], which finally leads to an energy crisis, cell death, and organ injury. A previous study showed that increasing concentrations of CDCA (100–1000 μM) influenced the impairment of mitochondrial membrane potential, decreasing mitochondrial dehydrogenase activity and enhancing mitochondrial permeabilization and swelling in mouse liver mitochondria [46]. In addition, studies also showed toxic effects in human colonic T84 cells when the CDCA level was at 500 μM [47] or in Caco-2 cells when the CDCA level was above 400 μM [48]. Indeed, in the present study, we found that when concentrations of CDCA were greater than 100 μM, there was a significant decrease in cell viability. However, conversely, 50 μM CDCA could increase the mitochondrial membrane potential and promote cell proliferation. These findings suggested that an appropriate concentration was very important for CDCA to play a role in promoting mitochondrial function in vitro.

Mitochondrial biogenesis is a complex process. In mammalian cells, the mitochondrion is an organelle that harbors its genome mtDNA, which encodes 22 tRNAs, 13 mRNAs, and 2 rRNAs. All 13 mRNAs of mtDNA encode 11 subunits and 2 ATP synthases, including complex I (ND1, ND2, ND3, ND4, ND4L, ND5, and ND6); subunit of complex III (CYTb); subunits of complex IV (COX1, COX2, and COX3); and subunits of ATP synthase (ATP6 and ATP8) [49]. Mitochondria. metabolic processes are responsible for the generation of ATP and ROS. Subunits of complexes I and IV are responsible for proton pumping in the mitochondria. ATP6 is a key component of the proton channel of the F1F0-ATPase complex. It is worth noting that the results of this study revealed that both the mtDNA-encoded 11 subunits of complexes and subunits of ATP synthase-related gene expression were downregulated by CDCA treatment. However, both the copy number of mtDNA and the level of intracellular ATP were enhanced by CDCA. It can be speculated that these results may reflect the negative feedback regulation in mitochondrial biogenesis-related gene expression or mitochondria with more efficient energy metabolism induced by CDCA, which needs further investigation.

The mitochondrial membrane potentials were often altered by the activities of respiratory chain complexes, which indicated the viability of cells [50]. In this study, an increased mitochondrial membrane potential in IPEC-J2 cells was observed after CDCA treatment. The increase in mitochondrial membrane potential may be due to the strongly increased efficiency of respiratory chain-mediated proton extrusion for the matrix. In general, normal cells conduct energy metabolism primarily through efficient mitochondrial oxidative phosphorylation (mtOXPHOS). The previous study showed that the enhancement of mtOXPHOS can lead to less electron leakage from the electron transport chain, and in turn, reduce the production of ROS in cells [51]. In the present study, the levels of intracellular ROS in the IPEC-J2 cells were significantly decreased by CDCA treatment, which may further reflect that CDCA could promote efficient mtOXPHOS. Furthermore, intracellular ROS overproduction will establish a vicious cycle of oxidative stress in the mitochondria, ultimately damaging mitochondrial and cellular proteins, lipids, and nucleic acids [52]. Our data clearly showed that CDCA could reduce oxidative stress in IECs and improve their antioxidant capacity, as reflected by the increased T-AOC and CAT activity and decreased MAD level, which could reduce oxidative stress-related mitochondrial dysfunction and cell damage. Mitochondria play a central role in the regulation of cell apoptosis, which is closely related to cell proliferation [53]. Several investigators have reported the different roles of BAs in intestinal cell proliferation, including stimulatory effects by taurine-conjugated cholic acid (TCA) [5] and glycochenodeoxycholic acid (GCDCA) [21], and inhibitory effects by deoxycholic acid (DCA) [5], ursodeoxycholic acid (UDCA) [22], and hyodeoxycholic acid (HDCA) [23]. However, these studies did not focus on the regulation of mitochondrial function by BAs, and it is unclear whether the regulation of cell proliferation by BAs depends on mitochondrial function. Therefore, even though we observed that CDCA promoted IEC proliferation related to improvement in mitochondrial function, it is necessary to further determine the role of mitochondrial function in the process of cell proliferation regulated by BA.

Apoptosis activation can be classically triggered by two distinct pathways: the extrinsic, or death receptor, and the intrinsic, or mitochondrial pathway. The decline in mitochondrial membrane potential is one of the characteristics of mitochondrial damage, which could induce the release of mitochondrial Cytochrome c into the cytosol, which further combines with Caspases to form a Caspase-activating complex [54]. Furthermore, signaling disruption of mitochondrial membrane potential could reflect increased mitochondrial membrane permeability and the occurrence of mitochondrial dysfunction—a pivotal and irreversible event in the apoptotic pathway [55]. Our results demonstrated that CDCA could increase mitochondrial membrane potential in IPEC-J2 cells, implying that CDCA may prevent IPEC-J2 cell apoptosis through the mitochondria-mediated pathway. In addition, the apoptosis process is regulated by two protein families: the Bcl-2 family, which is responsible for the initiation phase, and the Caspase family of proteases that are involved in the execution phase of apoptosis [56,57]. In the present study, CDCA did not affect the gene expression of Bcl-2, while it downregulated the gene expression of Caspase 2 and Caspase 8 in the IPEC-J2 cells, which can be explained from another perspective, namely that CDCA may promote cell proliferation by inhibiting cell apoptosis.

## 5. Conclusions

In summary, the present study combined flow cytometry and transcriptome analysis to reveal for the first time that in vitro, an appropriate concentration (50 μM) of CDCA could promote cell proliferation by accelerating cell cycle progression in the S and G2/M phases, inhibiting cell apoptosis and enhancing mitochondrial function by increasing mitochondrial membrane potential and antioxidant capacity, and decreasing intracellular ROS and MDA levels in IPEC-J2 cells. These findings provide a theoretical basis for promoting IEC proliferation and intestinal development in pigs by regulating intestinal BA metabolism.

## Data Availability

The RNA sequencing data were deposited in NCBI’s Gene Expression Omnibus (GEO) under the accession number GSE214153. The datasets used for the current study are available from the corresponding author upon reasonable request.

## Supplementary Materials

The following supporting information can be downloaded at: https://www.mdpi.com/article/10.3390/antiox11112285/s1, Table S1: Primers used for RT-qPCR analysis; Table S2: The raw data from RNA-seq analysis of CON and CDCA groups; Table S3: The node attributes table for the PPI network; Table S4: The node degree information for the PPI network; Table S5: Clusters of the PPI network; Table S6: The centrality degree of nodes centrality analysis for PPI network; Figure S1: Outlier box plots to identify biological outliers (A). All of the samples had a similar distribution of the data, and none were considered an outlier; The results of correlation analysis between samples (B). Results showed that biological replicates from the two groups could be well distinguished and the experimental design was reasonable. These results confirmed the high reproducibility of the sequencing data; Figure S2: Validation of transcriptomic results by RT-qPCR in IPEC-J2 cells. The 3 upregulated DEGs, 11 downregulated DEGs, and 6 unchanged genes observed by RNA-seq were selected for validation using RT-qPCR. All validated genes have similar trends in expression when comparing RNA-seq with RT-qPCR. # FDR < 0.05, * *p* < 0.05, ** *p* < 0.01, *** *p* < 0.001, versus CON group. IL-6, interleukin-6; CDK, cyclin-dependent kinase; ZO-1, zonula occludens-1; CLDN1, claudin 1; OGA, O-GlcNAcase; OGT, O-GlcNAc Transferase; Bax, BCL2-associated X; Bcl-2, B-cell lymphoma-2; FXR, farnesoid X receptor; LXRα, liver X receptor α; TGR5, G-coupled protein receptor; ND, NADH dehydrogenase subunit; COX, cytochrome c oxidase subunit; Cytb, cytochrome b; ATP, ATP synthase F0 subunit; Figure S3: The nodes centrality analysis for protein–protein interaction (PPI) network. Three centrality methods, including degree, closeness, and betweenness centrality, were used to explore the key genes in the PPI network. The detailed information for the centrality degree is shown in Table S6. Refs. [58,59,60,61,62,63,64,65,66,67,68] cited in Supplementary Materials.
