# Supplementary material for: Chenodeoxycholic Acid (CDCA) Promoted Intestinal Epithelial Cell Proliferation by Regulating Cell Cycle Progression and Mitochondrial Biogenesis in IPEC-J2 Cells
Source: Antioxidants (Basel). 2022 Nov 18;11(11):2285. doi: 10.3390/antiox11112285 (PMC9687205; doi:10.3390/antiox11112285)
Supplement: Supplementary file 1 [file antioxidants-11-02285-s001.zip › Supplementary Tables S1, S2 and Figures S1-S3.pdf]

# Supplementary Tables and Figures

## Table S1. Primers used for RT-qPCR analysis[1]

| Target gene | Forward primer (5'-3') | Reverse primer (5'-3') | References |
|---|---|---|---|
| CDK1 | TTTTCAGAGCTTTGGGCACTC | TTTTCGAGAGCSGATCCAAGC | [1] |
| p21 | AGGACCATGTGGACCTGTTG | TTAGGGCTTCCTCTTGGAGA | [2] |
| CDK4 | GCATCCCAATGTTGTCCG | GGGGTGCCTTGTCCAGATA | [3] |
| Caspase 2 | GCGACGCTGGCAAAGAG | CCTGAGGGCGAGGAACA | XM_005657720.3 |
| Caspase 3 | GCCATGGTGAAGAAGGAAAA | GTCCGTCTCAATCCCACAGT | [3] |
| Caspase 8 | TCCTGAGCCTGGACTACAT | CTCCTCCTCATTGGTTTCC | [3] |
| OGA | AAAAGCATGATGGCTTGCCT | CCACCCACCAGGTGAAGTTAA | [5] |
| OGT | AGCGGGACTCAATTACCCTTT | ATTGTCTGGCTCTTGTCTCCA | [5] |
| IL-6 | AATGTCGAGGCTGTGCAGATT | TGGTGGCTTTGTCTGGATTCT | NM_214399.1 |
| CLDN1 | AGCTGTGCATGGCCTCTTGT | CCAATGTCAATGGCAACACCCT | [7] |
| ZO-1 | CGGAACTATGACCATCGCCTAC | CTTCGGGATGTTGTCTGGAGTC | [7] |
| TGR5 | CCATGCACCCCTGTTGCT | GGTGCTGTTGGGTGTCATCTT | XM_013984487.2 |
| LXRα | AGAACAGATCCGCCTGAAGA | GGTCTGAAAAGGAGCGTCTG | XM_013994348.2 |
| FXR | TATGAACTCAGGCGAATGCCTGCT | ATCCAGATGCTCTGTCTCCGCAAA | [8] |
| Bax | CCGAAATGTTTGCTGACG | AGCCGATCTCGAAGGAAGT | [4] |
| Bcl-2 | ACCTGAATGACCACCTAGAGC | TCCGACTGAAGAGCGAAC | [4] |
| ND1 | TCAACCCTAGCAGAAACCAACGA | AAGAATATGGCGAAAGGTCGGCT | [10] |
| ND2 | TTTCCTAACACAAGCCACAGCCTC | ATGCCTTGGGTTACTTCTGGGACT | [10] |
| ND3 | AGCACGCCTCCCATTCTCAAT | TGCTAGGCTTGCTGCTAGTAGG | [11] |
| ND4 | TCGCCTATTCATCAGTAAGTCA | GGATTATGGTTCGGCTGTGTA | [11] |
| ATP6 | TACCACACTCATTCACACCCACCA | TGTTCCTTGTGGTAGAAAGTGGGC | [10] |
| ATP8 | TGCCACAACTAGATACATCC | GCTTGCTGGGTATGAGTAG | [11] |
| COX2 | TGGCTTACCCTTTCCAACTAGGCT | TTGGGCATCCATTGTGCTAGTGTG | [10] |
| COX3 | ACACCCGAATTAGGAGGTTGCTGA | TACGCCTAGTGCAATGGTGATGGA | [10] |
| Cytb | TCACACGATTCTTCGCCTTCCACT | TAGGGTTGTTGGATCCGGTTTCGT | [10] |
| D-loop | ACACACCCTATAACGCCTTGCC | GGGTAGGTGCCTGCTTTCGTAG | [11] |
| β-actin | ATGCTTCTAGACGGACTGCG | GTTTCAGGAGGCTGGCATGA | [9] |
| GAPDH | GCTTGTCATCAATGGAAAGG | CATACGTAGCACCAGCATCA | [6] |

[1] IL-6, interleukin-6; CDK, cyclin-dependent kinase; ZO-1, zonula occludens-1; CLDN1, claudin 1; GAPDH, glyceraldehyde-3-phosphate dehydrogenase; OGA, O-GlcNAcase; OGT, O-GlcNAc Transferase; Bax, BCL2-associated X; Bcl-2, B-cell lymphoma-2; FXR, farnesoid X receptor; LXRα, liver X receptor α; TGR5, G-coupled protein receptor; ND1, NADH dehydrogenase subunit 1; ND2, NADH dehydrogenase subunit 2; ND3, NADH dehydrogenase subunit 3; ND4, NADH dehydrogenase subunit 4; COX2, cytochrome c oxidase subunit 2; COX3, cytochrome c oxidase subunit 3; Cytb, cytochrome b; ATP6, ATP synthase F0 subunit 6; ATP8, ATP synthase F0 subunit 8.

**Table S2.** The raw data from RNA-Sequencing analysis of CON and CDCA groups

| Group | Sample ID | Raw reads | Clean reads | Total mapped | Multiple mapped | Uniquely mapped | GC content (%) | Q20(%) | Q30 (%) |
|---|---|---|---|---|---|---|---|---|---|
| CON | CON_3 | 65,466,558 | 64,675,190 | 61,746,328(95.47%) | 5,768,052(8.92%) | 55,978,276(86.55%) | 51.99 | 98.2 | 94.76 |
| | CON_2 | 69,271,904 | 68,540,858 | 65,644,376(95.77%) | 4,686,129(6.84%) | 60,958,247(88.94%) | 51.46 | 98.29 | 94.97 |
| | CON_1 | 61,354,956 | 60,811,650 | 58,355,295(95.96%) | 4,267,914(7.02%) | 54,087,381(88.94%) | 50.91 | 98.39 | 95.2 |
| CDCA | CDCA_3 | 65,356,232 | 64,745,300 | 61,984,695(95.74%) | 3,125,867(4.83%) | 58,858,828(90.91%) | 51.37 | 98.4 | 95.25 |
| | CDCA_2 | 61,757,660 | 61,112,694 | 58,433,389(95.62%) | 2,999,136(4.91%) | 55,434,253(90.71%) | 51.75 | 98.33 | 95.08 |
| | CDCA_1 | 60,683,302 | 60,088,126 | 57,502,899(95.7%) | 2,907,690(4.84%) | 54,595,209(90.86%) | 51.48 | 98.34 | 95.1 |

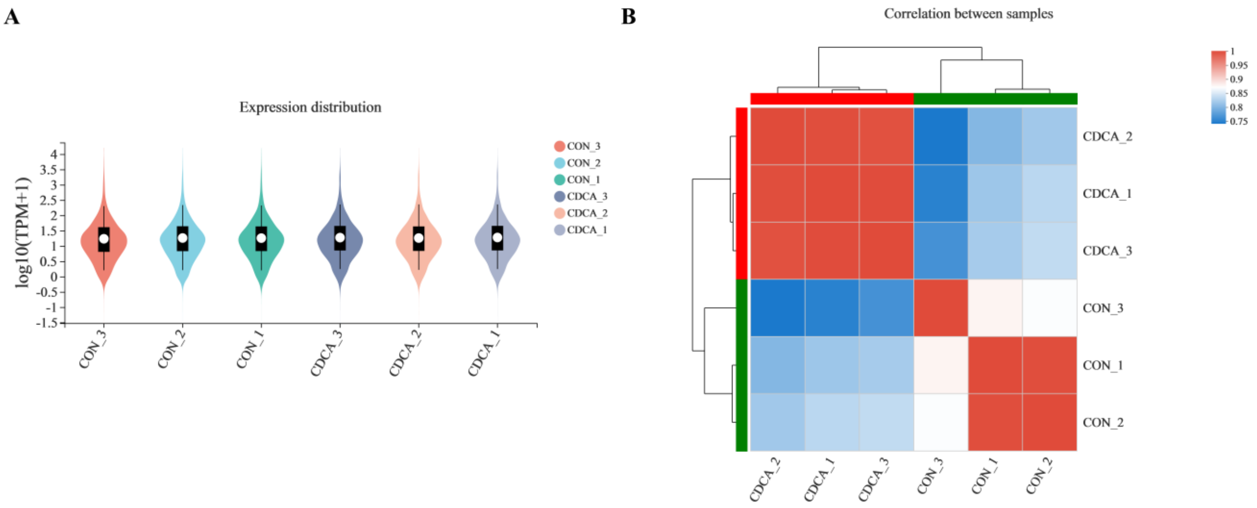


**Figure S1.** Outlier Box Plots to identify biological outliers (A). All the samples had a similar distribution of the data and none was considered an outlier; The results of correlation analysis between samples (B). Results showed that biological replicates from the two groups could be well distinguished and the experimental design was reasonable. These results confirmed the high reproducibility of the sequencing data.

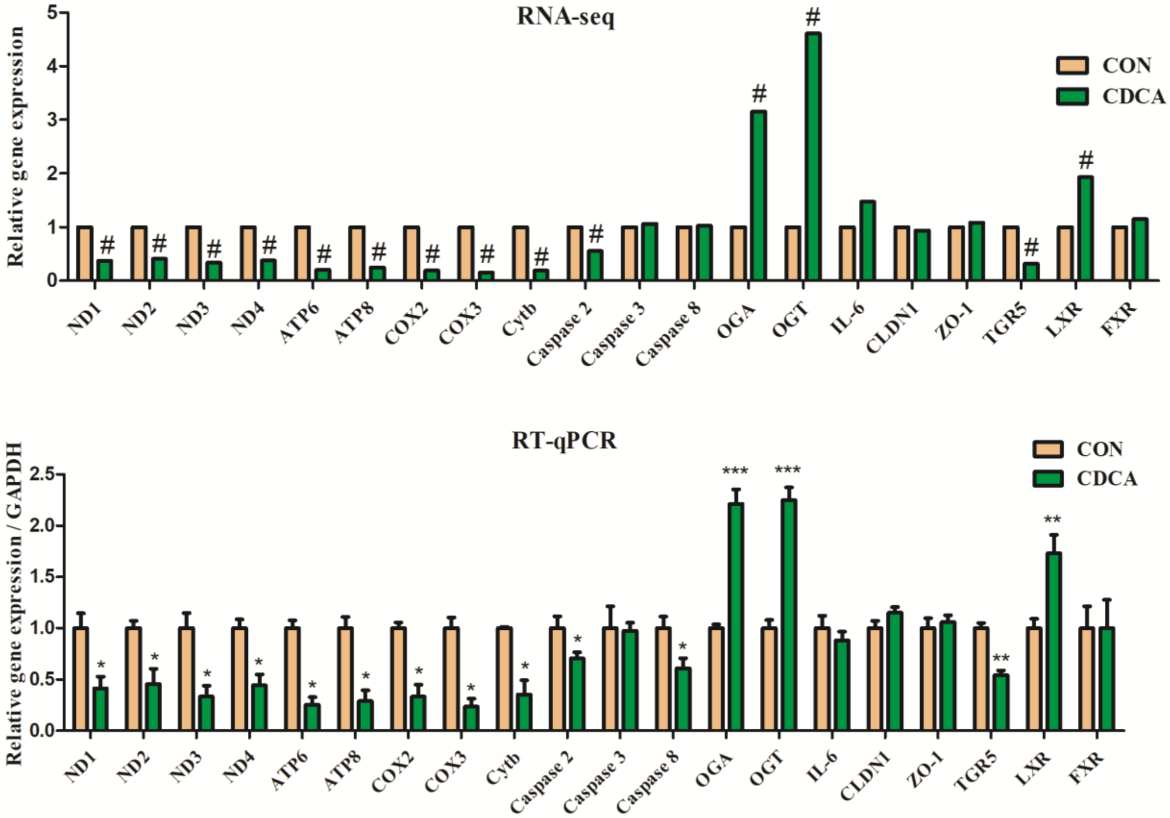


**Figure S2. Validation of transcriptomic results by RT-qPCR in IPEC-J2 cell.** The 3 upregulated DEGs, 11 downregulated DEGs, and 6 unchanged genes observed by RNA-seq were selected to validate using RT-qPCR. All validated genes have similar trends in expression when comparing RNA-seq with RT-qPCR. # FDR < 0.05, * $p < 0.05$, ** $p < 0.01$, *** $p < 0.001$, versus CON group. IL-6, interleukin-6; CDK, cyclin-dependent kinase; ZO-1, zonula occludens-1; CLDN1, claudin 1; OGA, O-GlcNAcase; OGT, O-GlcNAc Transferase; Bax, BCL2-associated X; Bcl-2, B-cell lymphoma-2; FXR, farnesoid X receptor; LXRα, liver X receptor α; TGR5, G-coupled protein receptor; ND, NADH dehydrogenase subunit; COX, cytochrome c oxidase subunit; Cytb, cytochrome b; ATP, ATP synthase F0 subunit.

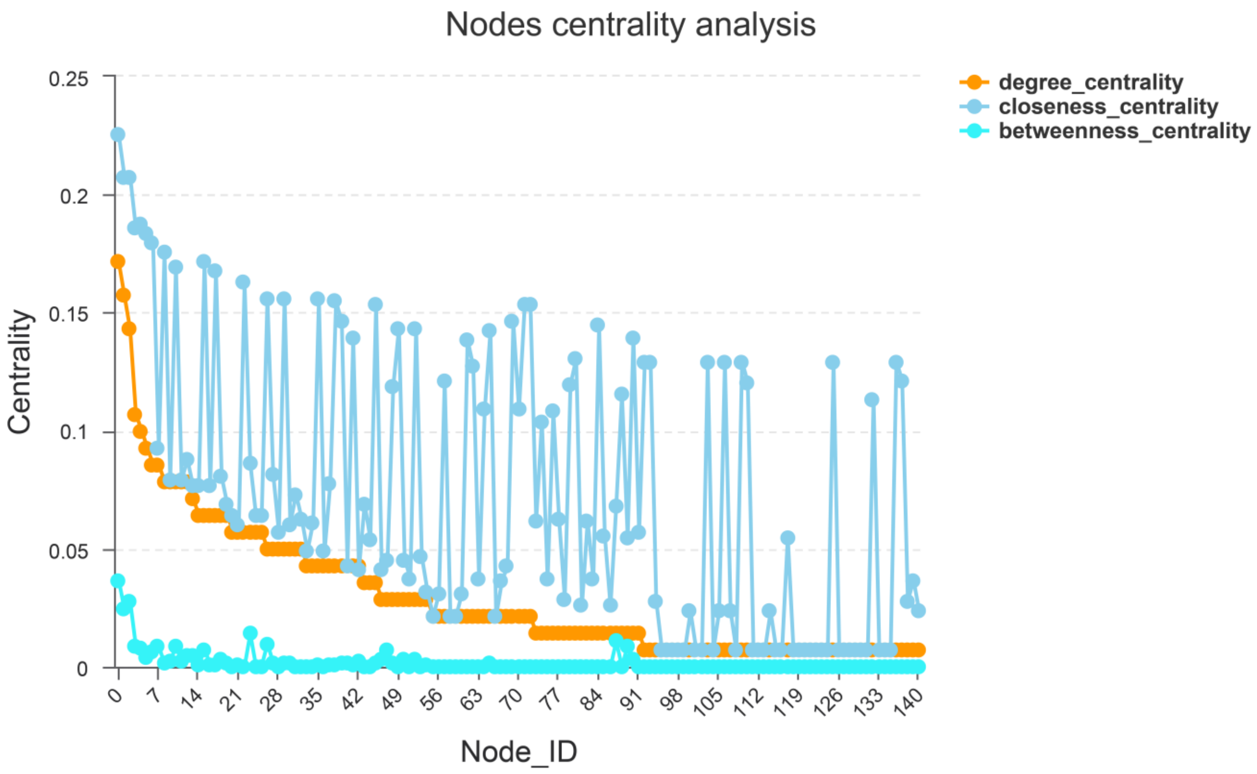


**Figure S3. The nodes centrality analysis for protein-protein interaction (PPI) network.** Three centrality methods including degree, closeness, and betweenness centrality were used to explore the key genes in the PPI network. The detailed information for the centrality degree is shown in Table S6.

## References


1. Lee J., You J., Lee G.S., Hyun S.H. & Lee E. (2013) Pig Oocytes With a Large Perivitelline Space Matured In Vitro Show Greater Developmental Competence After Parthenogenesis and Somatic Cell Nuclear Transfer. Molecular Reproduction and Development 80, 753-62.
2. Yin B.X., Ren H.Y., Cai H., Jiang Y.Q., Zhao S.H. & Wang H. (2020) Dynamics of cardiomyocyte and muscle stem cell proliferation in pig. Experimental Cell Research 388.
3. Ding H.X., Yang Y.Z., Wei S.L., Spicer L.J., Kenez A., Xu W., Liu Y. & Feng T. (2021) Influence of N-acetylcysteine on steroidogenesis and gene expression in porcine placental trophoblast cells. Theriogenology 161, 49-56.
4. Zhu L.H., Cai X., Guo Q., Chen X.L., Zhu S.W. & Xu J.X. (2013) Effect of N-acetyl cysteine on enterocyte apoptosis and intracellular signalling pathways' response to oxidative stress in weaned piglets. British Journal of Nutrition 110, 1938-47.
5. Shibutani M., Mori T., Miyano T. & Miyake M. (2015) Removal of O-GlcNAcylation is important for pig preimplantation development. Journal of Reproduction and Development 61, 341-50.
6. Cai L., Wei Z.X., Zhao X.M., Li Y.P., Li X.L. & Jiang X.R. (2022) Gallic acid mitigates LPS-induced inflammatory response via suppressing NF-kappa B signalling pathway in IPEC-J2 cells. Journal of Animal Physiology and Animal Nutrition 106, 1000-8.
7. Huang S.M., Wu Z.H., Li T.T., Liu C., Han D.D., Tao S.Y., Pi Y., Li N. & Wang J.J. (2020) Perturbation of the lipid metabolism and intestinal inflammation in growing pigs with low birth weight is associated with the alterations of gut microbiota. Science of the Total Environment 719.
8. Radtke J., Geissler S., Schutkowski A., Brandsch C., Kluge H., Duranti M.M., Keller S., Jahreis G., Hirche F. & Stangl G.I. (2014) Lupin protein isolate versus casein modifies cholesterol excretion and mRNA expression of intestinal sterol transporters in a pig model. Nutrition & Metabolism 11.
9. Lin M., Zhang B.L., Yu C.N., Li J.L., Zhang L., Sun H., Gao F. & Zhou G.H. (2014) L-Glutamate supplementation improves small intestinal architecture and enhances the expressions of jejunal mucosa amino acid receptors and transporters in weaning piglets. Plos One 9.
10. Weller M.M.D.C.A., Alebrante L., Campos P.H.R.F., Saraiva A., Silva B.A.N., Donzele J.L., et al. Effect of heat stress and feeding phosphorus levels on pig electron transport chain gene expression. Animal 2013;7(12):1985-93.
11. Jia Y, Song H, Gao G, Cai D, Yang X, Zhao R. Maternal betaine supplementation during gestation enhances expression of mtDNA-encoded genes through D-loop DNA hypomethylation in the skeletal muscle of newborn piglets. J Agric Food Chem 2015;63(46):10152-60.
